# Anethole Ameliorates Acetic Acid-Induced Colitis in Mice: Anti-Inflammatory and Antioxidant Effects

**DOI:** 10.1155/2022/9057451

**Published:** 2022-04-06

**Authors:** Maryam Ghasemi-Dehnoo, Amir Abbas Safari, Mohammad Rahimi-Madiseh, Zahra Lorigooini, Mohammad Taghi Moradi, Hossein Amini-Khoei

**Affiliations:** Medical Plants Research Center, Basic Health Sciences Institute, Shahrekord University of Medical Sciences, Shahrekord, Iran

## Abstract

Anethole has possessed anti-inflammatory and antioxidant responses in numerous studies. Oxidative stress has a pivotal role in the pathophysiology of colitis. The current study is designed to determine the effect of anethole on acetic acid-induced colitis in mice in view of its possible anti-inflammatory and antioxidant properties. In this study, 48 mice were grouped into 6 groups (*n* = 8), and colitis was induced with 0.2 ml of 7% acetic acid. Mice received intraperitoneally (i.p.) for 7 constant days normal saline and/or anethole at doses of 31.25, 62.5, 125, and 250 mg/kg, respectively. After treatments, the colon was dissected out, and histopathological changes, expression of inflammatory genes (IL-1*β*, TNF-*α,* and TLR4), and evaluation of malondialdehyde (MDA) levels and total antioxidant capacity (TAC) were assessed. The results showed that colitis is associated with edema and inflammatory responses in all layers and severe damage to the epithelium of the colon. Colitis causes a decrease in TAC, an increase in MDA levels, and an increase in inflammatory genes in the colon. Findings determined that anethole ameliorated the adverse effects of acetic acid-induced colitis in the colon. It is concluded that anethole, partially at least, possessed protective effects in acetic acid-induced colitis in mice through attenuation of oxidative stress and inflammatory response.

## 1. Introduction

Inflammatory bowel disease (IBD) is linked with diarrhea, fecal bleeding, abdominal pain, and additional colonic signs such as weakness, joint pain, and weight loss [1]. Two major forms of IBD are including ulcerative colitis (UC) and Crohn's disease (CD) [2, 3].

Abnormal immune response to commensal flora causes over activation of the innate immune system [4]. In this situation, neutrophils penetrated into the epithelium, leading to production of proinflammatory intermediaries such as cytokines, eicosanoids, and reactive oxygen species [4]. Throughout the inflammatory response, activation of toll-like receptors (TLRs) leads to creation of proinflammatory cytokines, including TNF-*α* and IL-1*β* in the epithelium [5]. It has been well-found that colitis is linked with increase in creation of lipid peroxidation products such as MDA and free radicals [6].

Now, immunosuppressive and anti-inflammatory drugs are commonly prescribed for management of IBD. These medications are not effective in all patients; furthermore, various side effects limit long time use of these drugs [7]. As a result, researchers are seeking for effective and safe agents for management of IBD. In this regard, in recent years, herbal and natural compounds have been very much considered by researchers [8].

Medicinal plants and their active compounds have various pharmacological effects, containing anti-inflammatory properties [9–13]. In this respect, earlier studies have demonstrated that medicinal plants and natural products diminished the inflammatory response in colitis [14–18]. Plenty evidence have exhibited that medicinal plants and their natural compounds possessed antioxidant activities [19, 20]. There are two anethole isomers in nature, including *Z* (cis-anethole) and *E* (trans-anethole) [21]. Trans-anethole is abundantly found in fennel, anise, and star anise, as well as in about 20 other plant species [22]. Preceding studies have been described numerous pharmacological possessions for anethole counting anti-inflammatory [23, 24], immune modulatory [25], anticancer [26] neuroprotective [27], wound healing [28], antidiabetes [29, 30], and skin protector [31] effects. Few studies have stated that anethole or extracts-contained anethole exerted protective effects in experimental colitis [32, 33]. Though, the particular mechanisms participated in these effects remains unclear. Therefore, the current trial is designed to assess the effect of anethole on acetic acid-induced colitis in mice focusing its probable anti-inflammatory and antioxidant possessions.

## 2. Materials and Methods

### 2.1. Animals

48 male NMRI mice were used for experiments. Mice (weighing between 25 and 30 g) were bought from Pasteur Institute (Tehran, Iran) and preserved in customary laboratory environment (21–24°C, 12-hour light/dark cycle, and free access to water and standard food).

### 2.2. Acetic Acid-Induced Colitis

To induce colitis, after one day of fasting, by intraperitoneal injection of ketamine (60 mg/kg) and xylazine (6.67 mg/kg), mice were sedated. 0.2 ml of 7% acetic acid was administered intrarectally (3 cm). Acetic acid was injected as a single dose. For the control group, in place of acetic acid, mice were similarly given 0.2 ml of saline phosphate buffer [34].

### 2.3. Study Design

Mice were arbitrarily distributed into 6 groups (8 mice in each group). Group 1 was considered as the control group (without induction of colitis), which received normal saline (1 ml/kg). In the groups 2–6, colitis induced and normal saline (1 ml/kg) or anethole (Sigma Aldrich, St. Louis, MO, USA) at doses of 31.25, 62.5, 125, and 250 mg/kg were administrated. Treatments were begun intraperitoneally (i.p.) one hour after the induction of colitis and persistent for one week.

After treatments, mice underwent deep anesthesia and euthanized with ketamine and xylazine. Colon tissues were dissected out and placed on the ice-cold surface, washed in PBS to eliminate fecal debris, and stored in formaldehyde for histopathological assessment. Furthermore, some pieces of colons tissues are directly engrossed in liquid nitrogen and saved at −70°C for valuation of gene expression of inflammatory genes, including TLR4, IL-1*β*, and TNF-*α.* Furthermore, malondialdehyde (MDA) levels and total antioxidant capacity (TAC) were assessed in the colon samples.

### 2.4. Histological Evaluations

Colon samples were dissected out, washed in PBS, fixed in 10% formaldehyde and stable in paraffin till processing. 5 *μ*m slices were prepared from every piece and set for staining with hematoxylin-eosin (H&E). 8 pieces from each group were considered for histopathological evaluations. In order to grading, from each sample, five sections were evaluated. The result of microscopic examinations was available based on the scoring system [35].

A blinded histopathologist examined sections in aspects of epithelial damage, epithelium thickness, edema, and infiltration of inflammatory cells using a light microscope equipped with a fitted Nikon camera.

### 2.5. Total Antioxidant Capacity (TAC) Assay

For dimension of total antioxidant capacity in the colon samples, ferric reducing/antioxidant power (FRAP) was used based on formerly approved method [36, 37]. In short, this method measured TAC by calculating the ability of reducing Fe^3+^ to Fe^2+^ using the FRAP assay kit (Naxifer™, Iran). FeSO_4_ is (100–1,000 *μ*M) considered as a standard in FRAP assay.

### 2.6. Malondialdehyde (MDA) Assay

The thiobarbituric acid (TBA) assay kit (Navand Salamat, Iran) was used for measurement of MDA levels. TBA reagent was mixed with colon homogenates to yield colored mixtures according to the manufacturer's etiquette. After centrifugation, the absorbance of supernatants was measured at 532 nm. We measured MDA concentrations based on the standard curve [38].

### 2.7. Real-Time PCR Analysis

RNX-plus isolation reagent according to the producer's directions was used for RNA extraction. RNA was computed using NanoDrop technologies. Real-time polymerase chain reaction (PCR) was performed for measurement of differences in the mRNA expression. After reverse transcription of mRNA with the PrimeScript RT reagent kit (Takara, Japan) according to the manufacturer's instruction, the qRT-PCR test was complete on a light cycler device (Rotor Gene Diagnostics) by the SYBR Premix Ex Taq technology (Takara). The thermal cycling platform design was 95°C for 30 s, followed by 45 cycles of denaturation for 5 s at 95°C, annealing step for 15 s at 60°C, and delay for 15 s at 72°C [39]. Melting curve analysis was used to admit whether all primers yield a single PCR creation. Beta 2-microglobulin (B2m) was amplified as a normalizer. The fold changes in expression of every target mRNA were measured in comparison to B2m based on 2^−ΔΔCt^ relative expression formula, as designated earlier by Arabi et al. [37].  Table 1 presents the primer sequences.

### 2.8. Statistical Analysis

Statistical analysis was prepared using version 21 of SPSS software. The Kolmogorov–Smirnov test was applied to evaluate the appropriateness for normal distribution. One-way ANOVA analysis followed by Tukey's post-hoc test was performed for data (means ± SEM) analyzing. *P* < 0.05 was deliberated as significant differences among groups.

## 3. Results

### 3.1. Anethole Mitigated the Histopathological Changes following Colitis

**Figure 1:**
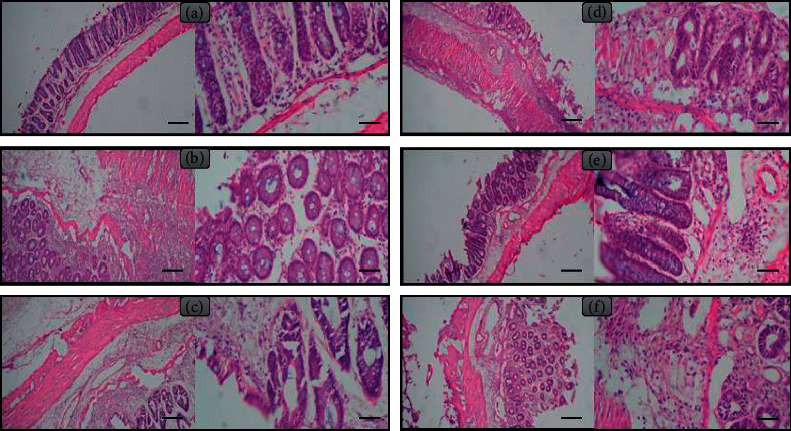
Illustrative features of histopathologic valuations delivered from H&E-stained colon sections (in each figure, left side is in × 10 and the right side in × 40 magnification). (a) Control. (b) Colitis. (c) Anethole at dose of 31.25 mg/kg. (d) Anethole at a dose of 62.5 mg/kg. (e) Anethole at dose of 125 mg/kg. (f) Anethole at a dose of 250 mg/kg.

As shown in Figure 1, edema, severe inflammation, inflammatory cell penetration, and injuries to the epithelium were noticed in the colitis group. We perceived that administration of anethole in a dose-dependent way diminished injury to the epithelium, penetration of inflammatory cells, and edema.

### 3.2. Anethole Augmented the Antioxidant Capacity in Colon Tissue

**Figure 2:**
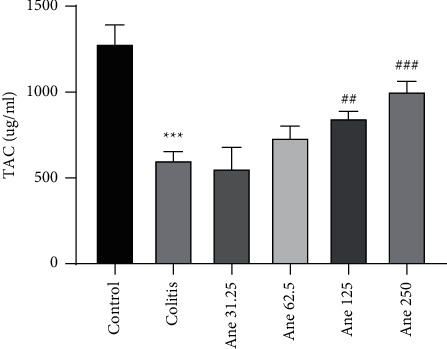
Total antioxidant capacity in the colon tissue. Data are expressed as the mean ± SEM (8 samples) and were analyzed by one-way ANOVA followed by Tukey's post-hoc test. ^*∗∗∗*^*P* < 0.001 in comparison with the control group and ^##^*P* < 0.01 and ^###^*P* < 0.001 in comparison with the colitis group (saline-treated). Ane, anethole; TAC, total antioxidant capacity.

As shown in Figure 2, colitis meaningfully reduced TAC of colon tissue in comparison to the control group (*P* < 0.001). Consequences showed that injection of anethole at doses of 125 and 250 mg/kg meaningfully augmented TAC of colon tissue in association with the saline-received colitis group (*P* < 0.01 and *P* < 0.001, one-to-one).

### 3.3. Anethole Declined the Malondialdehyde Level in the Colon Tissue

**Figure 3:**
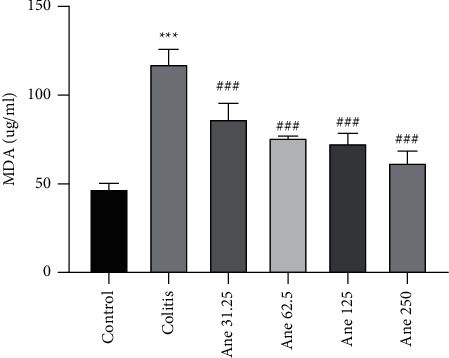
Malondialdehyde level in the colon in the experimental groups. Data are expressed as the mean ± SEM (8 samples) and were analyzed by one-way ANOVA followed by Tukey's post-hoc test. ^*∗∗∗*^*P* < 0.001 in comparison to the control group and ^###^*P* < 0.001 comparison with the colitis (saline-treated) group. Ane, anethole; MDA, malondialdehyde.

The finding displayed that the level of MDA in the colon samples meaningfully increased in the colitis group in relationship to the control group (*P* < 0.001, Figure 3). Administration of anethole at doses of 31.25, 62.5, 125, and 250 mg/kg pointedly diminished the level of MDA in comparison to the colitis (saline-treated) group (*P* < 0.001).

### 3.4. Anethole Condensed the Expression of Inflammatory Genes in the Colon

**Figure 4:**
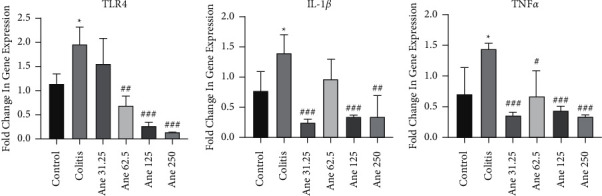
The gene expression of inflammatory associated cytokines in the colon. Values are expressed as the mean ± SEM (8 samples) and were analyzed by one-way ANOVA followed by Tukey's post-hoc test. ^*∗*^*P* < 0.05 in comparison to the control group and ^#^*P* < 0.05, ^##^*P* < 0.01, and ^###^*P* < 0.001 in relationship to the colitis group (saline-treated). Ane, anethole.

Based on the results (Figure 4), the gene expression of inflammatory mediators counting TNF-*α*, IL-1*β*, and TLR4 noticeably amplified in the colitis group in comparison to the control group (*P* < 0.05).

Injection of anethole at doses of 31.25 (*P* < 0.001), 62.5 (*P* < 0.05), 125 (*P* < 0.001), and 250 mg/kg (*P* < 0.001) meaningfully reduced the expression of TNF-*α* in relationship to the colitis (saline-treated) group.

In the case of the IL-1*β* gene expression, we found that anethole at doses of 31.25 (*P* < 0.001), 125 (*P* < 0.001), and 250 mg/kg (*P* < 0.01) considerably decreased the expression of IL-1*β* in comparison to the colitis (saline-treated) group.

Findings showed that anethole at doses of 62.5 (*P* < 0.01), 125 (*P* < 0.001), and 250 mg/kg (*P* < 0.001) meaningfully reduced the expression of TLR4 in comparison to colitis (saline-received) mice.

## 4. Discussion

In the current study, we established that the expression of inflammatory genes and MDA level considerably increased following colitis in the colon. In contrast, TAC significantly diminished in the colitis group in comparison to the control group. In histopathological appraisals, colitis was allied with edema, damage to the epithelium, and infiltration of inflammatory cells. Discoveries have demonstrated that anethole efficiently declined the abovementioned effects of colitis in the colon tissue, indicating that anethole exerted anti-inflammatory and antioxidant effects.

Prior research studies have validated that inflammatory response and oxidative stress have crucial roles in the development of colitis [4]. In colitis, damage to the epithelium increases the uptake of bacterial endotoxins like LPS (lipopolysaccharide) by the epithelium. TLR4 in the intestine wall identifies these LPSs [40]. The interface of LPS with TLR4 activates a signaling flow, which leads to the activation of NF-*κ*B. This phenomenon leads to creation of proinflammatory cytokines as well as TNF-*α* and IL-1*β* [41]. Ample indications have revealed that following colitis gene expression of inflammatory cytokines increased in the colon tissue [42, 43]. In this respect, clinical and preclinical research studies have exhibited that overproduction of oxidative stress indicators is involved in the pathophysiology of colitis [44, 45]. The presence of inflammation in mucosa leads to peroxidation of lipids which increased the levels of MDA and other markers of oxidative stress [41, 46]. It has been well-established that total antioxidant capacity decreases in the colon following colitis [47, 48]. In agreement with the aforesaid studies, we exhibited that the level of MDA meaningfully increased and TAC significantly diminished in the colitis group. Additionally, we found that colitis is associated with meaningfully increase in gene expression of inflammatory cytokines including TLR4, TNF-*α* and IL-1*β*.

Various studies have verified that some of the natural compounds, including caffeic acid [49], nerolidol [50], tricin [51], and also alpha-ketoglutarate [52], were effective in attenuating the inflammatory response in the colon following colitis. Trans-anethole is a phenylpropanoid structure which exist in the fennel, anise, and star anise plants [53, 54]. The anti-inflammatory, antioxidant, antimicrobial, and anticancer possessions of anethole have been proven in earlier published studies [55–58]. It has been shown that anethole exerted anti-inflammatory properties in numerous models of inflammatory states [55, 59–61]. Different studies have showed that anethole had no chronic toxicity effects (LD50 about 1820–5000 mg/kg). The doses used in this study are much lower than LD50 stated in previous studies [23, 28, 62]. Our consequences displayed that anethole considerably decreased the level of MDA, increased the TAC, and decreased the gene expression of inflammatory cytokines including TLR4, TNF*-α* and IL-1*β* in the colon tissue of the colitis group.

Numerous studies have established that intrarectal injection of acid acetic induce experimental colitis in rodents [63]. Colitis is associated with edema, damage to the colonic epithelium, and permeation of inflammatory cells including macrophages and neutrophils to the epithelium [63–65]. In the present study of infiltration of inflammatory cells, edemas in addition to epithelial lesions were observed in the colon tissue. The finding exhibited that anethole dose-dependently reduced edema, epithelial damage, and infiltration of inflammatory cells to the colon tissue.

Few studies have verified a protective effect for anethole or extracts-contained anethole in experimental colitis; however, the exact mechanisms that mediated this effect are unclear [32, 33]. In aspect of novelty of our study, it is important to declare that anethole, partially at least, through attenuation of oxidative stress (as decreased in the level of MDA and increased in the TAC) along with anti-inflammatory response (reduced gene expression of inflammatory cytokines) reversed contrary effects of colitis. In the other word, we demonstrated that anethole via its antioxidant and anti-inflammatory effects possessed beneficial effects in attenuation of experimental colitis. However, more studies are warranted to find more underlying mechanisms participated in the effects of anethole in colitis.

## 5. Conclusion

Generally, our finding confirmed that, possibly, anethole through attenuation of oxidative stress as well as alleviation of inflammatory response reduced the histopathological deviations of experimental colitis in the colon tissue.

## Figures and Tables

**Table 1:** Primer sequences.

Primer	Forward sequence	Reverse sequence
B2m	CTGCTACGTAACACAGTTCCACCC	CATGATGCTTGATCACATGTCTCG
IL-1*β*	GAAATGCCACCTTTTGACAGTG	TGGATGCTCTCATCAGGACAG
TLR4	ATGGCATGGCTTACACCACC	GAGGCCAATTTTGTCTCCACA
TNF-*α*	CTGAACTTCGGGGTGATCGG	GGCTTGTCACTCGAATTTTGAGA

## Data Availability

Our data are available during manuscript submission.

## References

[1] Haapamäki J., Turunen U., Roine R. P., Färkkilä M. A., Arkkila P. E. (2009). Impact of demographic factors, medication and symptoms on disease-specific quality of life in inflammatory bowel disease. *Quality of Life Research*.

[2] Murad H. A. S., Abdallah H. M., Ali S. S. (2016). Mentha longifolia protects against acetic-acid induced colitis in rats. *Journal of Ethnopharmacology*.

[3] Tekeli İ. O., Ateşşahin A., Sakin F., Aslan A., Çeribaşı S., Yipel M. (2019). Protective effects of conventional and colon-targeted lycopene and linalool on ulcerative colitis induced by acetic acid in rats. *Inflammopharmacology*.

[4] Wallace K. L., Zheng L.-B., Kanazawa Y., Shih D. Q. (2014). Immunopathology of inflammatory bowel disease. *World Journal of Gastroenterology*.

[5] Shaker M. E., Ashamallah S. A., Houssen M. E. (2014). Celastrol ameliorates murine colitis via modulating oxidative stress, inflammatory cytokines and intestinal homeostasis. *Chemico-Biological Interactions*.

[6] Liu D.-M., Zhou C.-Y., Meng X.-L., Wang P., Li W. (2018). Thymol exerts anti-inflammatory effect in dextran sulfate sodium-induced experimental murine colitis. *Tropical Journal of Pharmaceutical Research*.

[7] Sales-Campos H., Basso P., Alves V. (2014). Classical and recent advances in the treatment of inflammatory bowel diseases. *Brazilian Journal of Medical and Biological Research*.

[8] Bastaki S. M., Adeghate E., Amir N., Ojha S., Oz M. (2018). Menthol inhibits oxidative stress and inflammation in acetic acid-induced colitis in rat colonic mucosa. *American Journal of Tourism Research*.

[9] Rahaman M., Rakib A., Mitra S. (2021). The genus curcuma and inflammation: overview of the pharmacological perspectives. *Plants*.

[10] Sakib S. A., Tareq A. M., Islam A. (2021). Anti-inflammatory, thrombolytic and hair-growth promoting activity of the n-hexane fraction of the methanol extract of *Leea indica* leaves. *Plants*.

[11] Rakib A., Hasan M. S., Amin M. N., Mow T. R., Uddin M. M. N. (2018). Sedative, anxiolytic, antinociceptive, anti-inflammatory and antipyretic effects of a chloroform extract from the leaves of urena sinuata in rodents. *Journal of Applied Life Sciences International*.

[12] Dutta M., Nezam M., Chowdhury S. (2021). Appraisals of the Bangladeshi medicinal plant *Calotropis gigantea* used by folk medicine practitioners in the management of COVID-19: a biochemical and computational approach. *Frontiers in molecular biosciences*.

[13] Bari M. S., Khandokar L., Haque E. (2021). Ethnomedicinal uses, phytochemistry, and biological activities of plants of the genus Gynura. *Journal of Ethnopharmacology*.

[14] Pagano E., Venneri T., Lucariello G. (2021). Palmitoylethanolamide reduces colon cancer cell proliferation and migration, influences tumor cell cycle and exerts in vivo chemopreventive effects. *Cancers*.

[15] Pagano E., Romano B., Iannotti F. A. (2019). The non-euphoric phytocannabinoid cannabidivarin counteracts intestinal inflammation in mice and cytokine expression in biopsies from UC pediatric patients. *Pharmacological Research*.

[16] Pagano E., Capasso R., Piscitelli F. (2016). An orally active cannabis extract with high content in cannabidiol attenuates chemically-induced intestinal inflammation and hypermotility in the mouse. *Frontiers in Pharmacology*.

[17] Fernández J., Silván B., Entrialgo-Cadierno R. (2021). Antiproliferative and palliative activity of flavonoids in colorectal cancer. *Biomedicine & Pharmacotherapy*.

[18] Martínez V., Iriondo De-Hond A., Borrelli F., Capasso R., Del Castillo M. D., Abalo R. (2020). Cannabidiol and other non-psychoactive cannabinoids for prevention and treatment of gastrointestinal disorders: useful nutraceuticals?. *International Journal of Molecular Sciences*.

[19] Sinan K. I., Akpulat U., Aldahish A. A. (2003). LC-MS/HRMS analysis, anti-cancer, anti-enzymatic and anti-oxidant effects of boerhavia diffusa extracts: a potential raw material for functional applications. *Antioxidants*.

[20] Rahman M. M., Reza A. S. M. A., Khan M. A. (2021). Unfolding the apoptotic mechanism of antioxidant enriched-leaves of *Tabebuia pallida* (lindl.) miers in EAC cells and mouse model. *Journal of Ethnopharmacology*.

[21] Aprotosoaie A. C., Costache I.-I., Miron A. (2016). Anethole and its role in chronic diseases. *Advances in Experimental Medicine & Biology*.

[22] Kang N. H., Mukherjee S., Min T., Kang S. C., Yun J. W. (2018). Trans-anethole ameliorates obesity via induction of browning in white adipocytes and activation of brown adipocytes. *Biochimie*.

[23] Freire R. S., Morais S. M., Catunda-Junior F. E. A., Pinheiro D. C. S. N. (2005). Synthesis and antioxidant, anti-inflammatory and gastroprotector activities of anethole and related compounds. *Bioorganic & Medicinal Chemistry*.

[24] Kang P., Kim K. Y., Lee H. S., Min S. S., Seol G. H. (2013). Anti-inflammatory effects of anethole in lipopolysaccharide-induced acute lung injury in mice. *Life Sciences*.

[25] Siddikuzzaman G. V., Berlin G. V. M. (2013). Evaluation of immunomodulatory and antitumor activity of alltransretinoic acid (ATRA) in solid tumor bearing mice. *Immunopharmacology and Immunotoxicology*.

[26] Chen A. Y., Kim S. E., Houtrow A. J., Newacheck P. W. (2010). Prevalence of obesity among children with chronic conditions. *Obesity*.

[27] Ryu S., Seol G. H., Park H., Choi I.-Y. (2014). Trans-anethole protects cortical neuronal cells against oxygen-glucose deprivation/reoxygenation. *Neurological Sciences*.

[28] Malveira Cavalcanti J., Henrique Leal-Cardoso J., Leite Diniz L. R. (2012). The essential oil of Croton zehntneri and trans-anethole improves cutaneous wound healing. *Journal of Ethnopharmacology*.

[29] Dongare V., Kulkarni C., Kondawar M., Magdum C., Haldavnekar V., Arvindekar A. (2012). Inhibition of aldose reductase and anti-cataract action of trans-anethole isolated from *Foeniculum vulgare* mill. fruits. *Food Chemistry*.

[30] Sheikh B. A., Pari L., Rathinam A., Chandramohan R. (2015). Trans-anethole, a terpenoid ameliorates hyperglycemia by regulating key enzymes of carbohydrate metabolism in streptozotocin induced diabetic rats. *Biochimie*.

[31] Galicka A., Krętowski R., Nazaruk J., Cechowska-Pasko M. (2014). Anethole prevents hydrogen peroxide-induced apoptosis and collagen metabolism alterations in human skin fibroblasts. *Molecular and Cellular Biochemistry*.

[32] Rezayat S. M., Dehpour A.-R., Motamed S. M. (2018). Foeniculum vulgare essential oil ameliorates acetic acid-induced colitis in rats through the inhibition of NF-*κ*B pathway. *Inflammopharmacology*.

[33] Alshahrani A., Ali A. (2022). Pre-clinical safety and efficacy evaluation of herbal nanoemulsion based formulation for treating inflammatory bowel disease. *Journal of AOAC International*.

[34] Gupta R. A., Motiwala M. N., Dumore N. G., Danao K. R., Ganjare A. B. (2015). Effect of piperine on inhibition of FFA induced TLR4 mediated inflammation and amelioration of acetic acid induced ulcerative colitis in mice. *Journal of Ethnopharmacology*.

[35] Neurath M. F., Fuss I., Kelsall B. L., Stüber E., Strober W. (1995). Antibodies to interleukin 12 abrogate established experimental colitis in mice. *Journal of Experimental Medicine*.

[36] Benzie I. F. F., Strain J. J. (1996). The ferric reducing ability of plasma (FRAP) as a measure of “antioxidant power”: the FRAP assay. *Analytical Biochemistry*.

[37] Arabi M., Nasab S. H., Lorigooini Z. (2021). Auraptene exerts protective effects on maternal separation stress-induced changes in behavior, hippocampus, heart and serum of mice. *International Immunopharmacology*.

[38] Ohkawa H., Ohishi N., Yagi K. (1979). Assay for lipid peroxides in animal tissues by thiobarbituric acid reaction. *Analytical Biochemistry*.

[39] Nouri A., Hashemzadeh F., Soltani A., Saghaei E., Amini-Khoei H. (2020). Progesterone exerts antidepressant-like effect in a mouse model of maternal separation stress through mitigation of neuroinflammatory response and oxidative stress. *Pharmaceutical Biology*.

[40] Miao F., Shan C., Shah S. A. H. (2021). The protective effect of walnut oil on lipopolysaccharide-induced acute intestinal injury in mice. *Food Sciences and Nutrition*.

[41] Mahmoud T. N., El-Maadawy W. H., Kandil Z. A., Khalil H., El-Fiky N. M., El Alfy T. S. M. A. (2021). Canna x generalis L.H. Bailey rhizome extract ameliorates dextran sulfate sodium-induced colitis via modulating intestinal mucosal dysfunction, oxidative stress, inflammation, and TLR4/NF-*κ*B and NLRP3 inflammasome pathways. *Journal of Ethnopharmacology*.

[42] Yin Q., Pi X., Jiang Y. (2021). An immuno-blocking agent targeting IL-1*β* and IL-17A reduces the lesion of DSS-induced ulcerative colitis in mice. *Inflammation*.

[43] Zhou Y., Zhong B., Min X. (2021). Therapeutic potential of isobavachalcone, a natural flavonoid, in murine experimental colitis by inhibiting NF-*κ*B p65. *Phytotherapy Research*.

[44] Balmus I., Ciobica A., Trifan A., Stanciu C. (2016). The implications of oxidative stress and antioxidant therapies in inflammatory bowel disease: clinical aspects and animal models. *Saudi Journal of Gastroenterology*.

[45] Zhu H., Li Y. R. (2012). Oxidative stress and redox signaling mechanisms of inflammatory bowel disease: updated experimental and clinical evidence. *Experimental Biology and Medicine*.

[46] Zhang H., Deng A., Zhang Z. (2016). The protective effect of epicatechin on experimental ulcerative colitis in mice is mediated by increasing antioxidation and by the inhibition of NF-*κ*B pathway. *Pharmacological Reports*.

[47] Koutroubakis I. E., Malliaraki N., Dimoulios P. D., Karmiris K., Castanas E., Kouroumalis E. A. (2004). Decreased total and corrected antioxidant capacity in patients with inflammatory bowel disease. *Digestive Diseases and Sciences*.

[48] Nikkhah-Bodaghi M., Darabi Z., Agah S., Hekmatdoost A. (2019). The effects of *Nigella sativa* on quality of life, disease activity index, and some of inflammatory and oxidative stress factors in patients with ulcerative colitis. *Phytotherapy Research*.

[49] Khan M. N., Lane M. E., McCarron P. A., Tambuwala M. M. (2018). Caffeic acid phenethyl ester is protective in experimental ulcerative colitis via reduction in levels of pro-inflammatory mediators and enhancement of epithelial barrier function. *Inflammopharmacology*.

[50] Bastaki S. M. A., Amir N., Adeghate E., Ojha S. (2021). Nerolidol, a sesquiterpene, attenuates oxidative stress and inflammation in acetic acid-induced colitis in rats. *Molecular and Cellular Biochemistry*.

[51] Li X.-X., Chen S.-G., Yue G. G.-L. (2021). Natural flavone tricin exerted anti-inflammatory activity in macrophage via NF-*κ*B pathway and ameliorated acute colitis in mice. *Phytomedicine*.

[52] Tian Q., Bravo Iniguez A., Sun Q., Wang H., Du M., Zhu M. J. (2021). Dietary alpha-ketoglutarate promotes epithelial metabolic transition and protects against DSS-induced colitis. *Molecular Nutrition & Food Research*.

[53] Goswami N., Chatterjee S. (2014). Assessment of Free Radical Scavenging Potential and Oxidative DNA Damage Preventive Activity of Trachyspermum Ammi L.(Carom) and Foeniculum Vulgare Mill. (Fennel) seed extracts. *BioMed Research International*.

[54] Ritter A. M. V., Domiciano T. P., Verri W. A. (2013). Antihypernociceptive activity of anethole in experimental inflammatory pain. *Inflammopharmacology*.

[55] da Rocha B. A., Ritter A. M. V., Ames F. Q. (2017). Acetaminophen-induced hepatotoxicity: preventive effect of trans anethole. *Biomedicine & Pharmacotherapy*.

[56] Ahmed S., Khan H., Aschner M., Mirzae H., Küpeli Akkol E., Capasso R. (2020). Anticancer potential of furanocoumarins: mechanistic and therapeutic aspects. *International Journal of Molecular Sciences*.

[57] Küpeli Akkol E., Genç Y., Karpuz B., Sobarzo-Sánchez E., Capasso R. (2020). Coumarins and coumarin-related compounds in pharmacotherapy of cancer. *Cancers*.

[58] Chainy G. B., Manna S. K., Chaturvedi M. M., Aggarwal B. B. (2000). Anethole blocks both early and late cellular responses transduced by tumor necrosis factor: effect on NF-*κ*B, AP-1, JNK, MAPKK and apoptosis. *Oncogene*.

[59] Domiciano T. P., Dalalio M. M. d. O., Silva E. L. (2013). Inhibitory effect of anethole in nonimmune acute inflammation. *Naunyn-Schmiedeberg’s archives of pharmacology*.

[60] Wisniewski-Rebecca E. S., Rocha B. A., Wiirzler L. A. M., Cuman R. K. N., Velazquez-Martinez C. A., Bersani-Amado C. A. (2015). Synergistic effects of anethole and ibuprofen in acute inflammatory response. *Chemico-Biological Interactions*.

[61] Moradi J., Abbasipour F., Zaringhalam J. (2014). Anethole, a medicinal plant compound, decreases the production of pro-inflammatory TNF-*α* and IL-1*β* in a rat model of LPS-induced periodontitis. *Iranian Journal of Pharmaceutical Research: IJPR*.

[62] Raman S., Asle-Rousta M., Rahnema M. (2020). Protective effect of fennel, and its major component trans-anethole against social isolation induced behavioral deficits in rats. *Physiology International*.

[63] Gupta R. A., Motiwala M. N., Mahajan U. N., Sabre S. G. (2018). Protective effect of Sesbania grandiflora on acetic acid induced ulcerative colitis in mice by inhibition of TNF-*α* and IL-6. *Journal of Ethnopharmacology*.

[64] Popov S. V., Markov P. A., Nikitina I. R., Petrishev S., Smirnov V., Ovodov Y. S. (2006). Preventive effect of a pectic polysaccharide of the common cranberry *Vaccinium oxycoccos* L. on acetic acid-induced colitis in mice. *World Journal of Gastroenterology*.

[65] Al-Rejaie S. S., Abuohashish H. M., Al-Enazi M. M., Al-Assaf A. H., Parmar M. Y., Ahmed M. M. (2013). Protective effect of naringenin on acetic acid-induced ulcerative colitis in rats. *World Journal of Gastroenterology*.

